# Factors associated with post-acute discharge location after hospital stay: a cross-sectional study from a Swiss hospital

**DOI:** 10.1186/s12913-019-4101-6

**Published:** 2019-05-08

**Authors:** Bettina M. Zimmermann, Insa Koné, Michael Rost, Agnes Leu, Tenzin Wangmo, Bernice S. Elger

**Affiliations:** 1 Institute for Biomedical Ethics, University of Basel, Bernoullistrasse 28, 4056 Basel, Switzerland; 2 Careum Research, Department Health Sciences, Kalaidos University of Applied Sciences, Pestalozzistrasse 3, 8032 Zürich, Switzerland

**Keywords:** Subacute care, Diagnosis-related groups, Acute and transitional care, Switzerland

## Abstract

**Background:**

In 2012, Switzerland introduced the diagnosis-related group hospital payment system. Fearing that vulnerable patients may be discharged early, Acute and Transitional Care (ATC) was introduced to address the nursing care of patients who no longer needed an acute hospital stay. ATC is more costly for patients when compared to other discharge options like rehabilitation while providing less rehabilitative services. This study investigates factors associated with the place of discharge for patients in need of care.

**Methods:**

Data was collected from 660 medical records of inpatients 50 years and older of the municipal hospital Triemli in Zurich, Switzerland. We used stepwise logistic regression to identify factors associated with their discharge into ATC or rehabilitation.

**Results:**

Older patients with higher Delirium Observation Scale (DOS), lack of supplementary health insurance, resuscitation order and a lower social network were more likely to be discharged into ATC than rehabilitation.

**Conclusions:**

The association of supplementary health insurance and social network with discharge into ATC or rehabilitation is problematic because patients that are already vulnerable from a financial and social perspective are potentially discharged into a more costly and less rehabilitative post-acute care facility.

**Electronic supplementary material:**

The online version of this article (10.1186/s12913-019-4101-6) contains supplementary material, which is available to authorized users.

## Background

Switzerland has a compulsory social health insurance system that covers a comparatively high basket of care types including costs for inpatient care. The introduction of an inpatient reimbursement system based on diagnosis-related groups in Switzerland (SwissDRG) in 2012 changed the incentive structures of the healthcare system in various ways. Namely, it meant to incentivize the reduction of hospital stays, higher productivity, general cost awareness, stronger structures and process quality, increased transparency and reduced medical training activities. Experts pointed out that misdirected incentives lead to disadvantages for vulnerable groups, e.g. inadequate reflection of chronically-ill and elderly patients within the new tariff structure SwissDRG [1, 2]. To overcome this risk, Switzerland introduced a new category of post-acute care services, the Acute and Transitional Care (ATC). ATC should temporarily take care of inpatients who no longer need to stay in acute hospital units but still require nursing care before returning home [3]. Similar post-acute transitional care units have been implemented in different national healthcare systems [4–6].

The mandatory basic health insurance covers inpatient hospital stays and also most outpatient and emergency treatments as determined by the Federal Act on Health Insurance (KVG, SR 832.10) of 18 March 1994. In addition, Swiss residents can purchase supplementary health insurances for additional treatments or special inpatient conditions, e.g. single room or chief physician treatment [7, 8].

The legal framework in Switzerland foresees that ATC is used directly following the acute hospital stay, is prescribed by a doctor at the hospital and lasts a maximum of 14 days (Art. 25a KVG). The detailed implementation of ATC is in the responsibility of the cantons (states) since inpatient facilities in Switzerland are organized at the cantonal level [9]. In the canton of Zurich, ATC is provided as inpatient care by regional nursing homes. Nursing care is covered by health insurance without previous cost approval, but hotel costs (i.e., room and board) must be paid by the patient [10]. The extent of additional offers like physiotherapy and ergotherapy are not standardized but need to be prescribed separately in ATC, as opposed to inpatient rehabilitation, which is another option for patients who have not yet regained their former abilities.

The aim of ATC is to reverse health impairments in order to return to an independent state post injury or disease. Rehabilitation has a similar aim, but the patient’s deficit must be specifically defined in order to have recovery aims that can be reached during rehabilitation. To be admitted to a rehabilitation clinic, patients need an advance cost approval from their health insurance. Besides diagnosis and handicap, the need, capability, and potential for rehabilitation are assessed for each patient by both the hospital physician and the health insurance [11]. If cost approval is obtained, rehabilitation costs are covered by health insurance to the same extent as acute hospital care but without any additional hotel costs. This means that from the patient’s perspective, ATC is more expensive than acute hospital care or rehabilitation, while the services offered are probably less comprehensive [12].

The recent changes in hospital reimbursement and discharge systems due to the introductions of DRG and ATC in Switzerland thus lead to new situations for patients, as in other countries worldwide [4]. We thus investigate where inpatients in need of care after acute hospital stay were discharged, and examine factors associated with discharge to ATC versus rehabilitation using quantitative analysis of medical records.

## Methods

This study is part of the SNF-funded project “Inpatient outpatient transition in the era of DRGs: the legal framework and current practice” and presents a quantitative analysis of medical records data from inpatients in need of post-acute care after an inpatient stay at Zurich municipal hospital Triemli. The cantonal ethics committee Zurich approved the study (No 2015–0350). Inpatients hospitalized between April and August 2016 for which the social workers in the hospital organized post-acute care were included. The hospital’s social service accompanies and advises the referral to places of discharge like rehabilitation or ATC in accordance with the recommendations of physicians and nurses, as well as the wishes of patients and their relatives. The time period of 5 months for data collection was chosen for resource reasons. Patient recruitment came from 14 social workers who had examined and supported patients in need of care post hospital stay, and we assessed their medical records. Only patients older than 50 years of age were included because we considered that the issue of premature discharge might be potentially more harmful to these patients.

We extracted data from full electronic medical records using a data extraction sheet, comprising data on socio-demographic variables, social network of the patient (relatives, friends, neighbors etc.), living conditions, health insurance status, diagnoses, medications, length of hospital stay (LOS), resuscitation status, scores measuring the need of care (e.g. Delirium Observation Scale (DOS), Braden), and others. The DOS [13] measures the state of consciousness of a patient and is routinely assessed by nurses. A lower DOS score indicates a higher state of consciousness. The Braden scale [14] is a risk assessment tool for pressure ulcers and is also routinely assessed by nurses. For care needs, nurses’ documentation on patients’ needs in different aspects of daily activities (mobility, care support, etc.) was used to retrospectively attribute a Katz score [15] at discharge.

We computed a medical social support sum score which is based on the validated Berkman-Syme Social Network Index [16]. The sum score consists of four dimensions, each of them scored 1 (yes) or 0 (no): (1) marital status, (2) the existence of living parents or children, (3) other relatives like siblings, grandchildren or cousins, and (4) other contacts like neighbors and friends. Accordingly, the sum score ranges from 0 to 4.

The data was manually entered into and analyzed using IBM SPSS Statistics 24.0 (SPSS Inc., Chicago, IL). We report group comparisons between ATC and rehabilitation as well as general sample data descriptively. Independent factors associated with place of discharge are determined by using forward stepwise logistic regression analysis. Predictors entered the model if alpha  < .20 and were removed if alpha  > .30. The stepwise method is useful for our exploratory study because the associations between the possible explanatory variables and the dependent variable (e.g. place of discharge) are not well understood due to the only recent introduction of ATC in Switzerland and stepwise selection provides a screening of the candidate variables [17]. Odds ratio and 95% confidence intervals were estimated in order to determine the magnitude of the association between the explanatory variables and the place of discharge. For each regression, we report the final model, namely the last step for which the included variable significantly increased the accuracy of the model (*p* < .05).

The following variables were included in the regression analyses: age (continuous variable), gender (dichotomous variable: male vs. female), Katz index (ranging from 0 to 6), DOS (ranging from 0 to 13), number of prescribed medication, number of diagnoses, social support sum score, living conditions (dichotomous: alone vs. not alone), health insurance (dichotomous: presence vs. absence of supplementary health insurance), “d*o not resuscitate”* (DNR) order (dichotomous: presence vs. absence). Patients with a positive DNR status do not want to be reanimated in case of a cardiac arrest [18, 19].

We first compared the patients discharged to ATC with those discharged to rehabilitation (regression I). Second, to get a deeper insight, we additionally performed regression analyses comparing patients discharged to rehabilitation versus all other places of discharge (regression II), as well as patients discharged to ATC versus all other places of discharge (regression III).

## Results

Descriptive findings of the variables collected from medical records are presented in Table 1 for patients discharged to ATC (*n* = 161), to rehabilitation (*n* = 262), and the mean value over the whole data set (*n* = 660, see Additional file 1 data for other places of discharge represented in our dataset). ATC patients were older (mean years of age 82.9 vs. 73.9) and a greater proportion was female (68.3% vs. 51.1%) as compared to patients discharged into rehabilitation. The health status was similar for both patient groups at the moment of hospital discharge, except that ATC patients had a higher DOS (1.7 vs. 0.8). ATC patients were more often living alone prior to hospitalization (62.4% vs. 48.8%) and were less likely to have supplementary health insurance compared to rehabilitation patients (18.6% vs. 34.1%). Inpatients discharged into rehabilitation, in turn, had a longer LOS (16.9 vs. 13.0 days) and more rarely had a *DNR* order (28.6% vs. 62.5%).

**Table 1:** Descriptive statistics of variables

Category	Variable	Specification	Mean (SD) / Percentage		
			ATC (*n* = 161)	Reha (*n* = 262)	All (*n* = 660)
Demographics	Age (*n* = 660)	Years	82.9 (10.0)	73.9 (10.5)	77.46 (11.1)
	Gender (*n* = 660)	% female	68.3%	51.1%	59.1%
Health status	Independence (*n* = 660)	Katz index	3.5 (1.7)	4.0 (1.7)	3.8 (1.8)
	Level of delirium (*n* = 511)	DOS	1.7 (2.0)	0.8 (1.2)	1.5 (2.1)
	Skin issues (*n* = 348)	Braden scale	17.7 (3.0)	17.9 (3.2)	17.8 (3.2)
	Medication (*n* = 660)	No of prescribed medication	8.6 (3.6)	8.7 (3.9)	8.7 (3.8)
	Diagnoses (*n* = 627)	No of diagnoses	9.6 (5.0)	10.2 (6.0)	9.9 (5.9)
Social support	Social support sum score (*n* = 660)	0 (no social network) to 4 (rich social network)	1.7 (0.8)	1.9 (0.8)	1.9 (0.8)
	Living conditions (*n* = 612)	% living alone	62.4%	48.8%	52.9%
	Health insurance (*n* = 658)	% with supplementary health insurance	18.6%	34.1%	29.3%
Hospitalization	LOS (*n* = 660)	Days	13.0 (6.9)	16.9 (10.3)	14.9 (9.7)
	DNR order (*n* = 546)	% with DNR order	62.5%	28.6%	46.7%

*Note*. *DNR* Do Not Resuscitate. *DOS* Delirium Observation Scale

First, regression analysis comparing discharge between ATC and rehabilitation (regression I) showed that the younger the patients were and the lower their DOS was (indicating a higher state of consciousness) the more likely they were of being sent into rehabilitation. Furthermore, the absence of DNR, the presence of supplementary health insurance and a higher social support sum score were factors predicting discharge into rehabilitation rather than ATC (Table 2).

**Table 2:** Factors associated with place of discharge for patients released to ATC or rehabilitation

Regression I: Discharge to ATC or rehabilitation (*n* = 259)			Regression II: Discharge to rehabilitation or other (*n* = 383)			Regression III: Discharge to ATC or other (*n* = 383)		
Variable	Odds ratio	95% CI	Variable	Odds ratio	95% CI	Variable	Odds ratio	95% CI
Age	0.93 ***	(0.90; 0.96)	Age	0.97 *	(0.94; 0.99)	Age	1.08 ***	(1.05; 1.11)
DNR	2.50 **	(1.36; 4.62)	DNR	2.39 ***	(1.47; 3.89)	Supplementary health insurance	0.34 ***	(0.20; 0.58)
DOS	0.60 ***	(0.47; 0.77)	DOS	0.60 ***	(0.50; 0.73)			
Social support sum score	1.49 *	(1.01; 2.19)	Gender	1.70 *	(1.06; 2.75)			
Supplementary health insurance	2.52 **	(1.31; 4.82)						
Hosmer–Lemeshow test: *P* = 0.730			Hosmer–Lemeshow test: *P* = 0.836			Hosmer–Lemeshow test: *P* = 0.486		

*Note*. Stepwise logistic regression. The binary dependent variable in this analysis is the place of discharge. Significance level for inclusion in the model was *p* = .20. Predictive significance levels: * *p* < .05, ** *p* < .01, *** *p* < 0.001. Hosmer–Lemeshow tests indicate that the overall model fit is good. Reference groups for dichotomous independent variables: DNR – 0 = No; insurance – 0 = public; gender – 0 = female. Reference groups for dichotomous dependent variables: rehabilitation = 1. *DOS* Delirium Observation Screening Scale, *DNR* Do Not Resuscitate

Second, we additionally compared each group to the whole rest of the data set (regressions II & III, Table 2). Again, those who were younger and those with lower DOS were more likely to be released into rehabilitation. Additionally, male gender and the absence of a DNR order were associated with discharge into rehabilitation. The older the patients were, the more likely they were to be discharged into ATC, and also the absence of supplementary health insurance was a predictor for discharge into ATC rather than any other place.

**Fig. 1:**
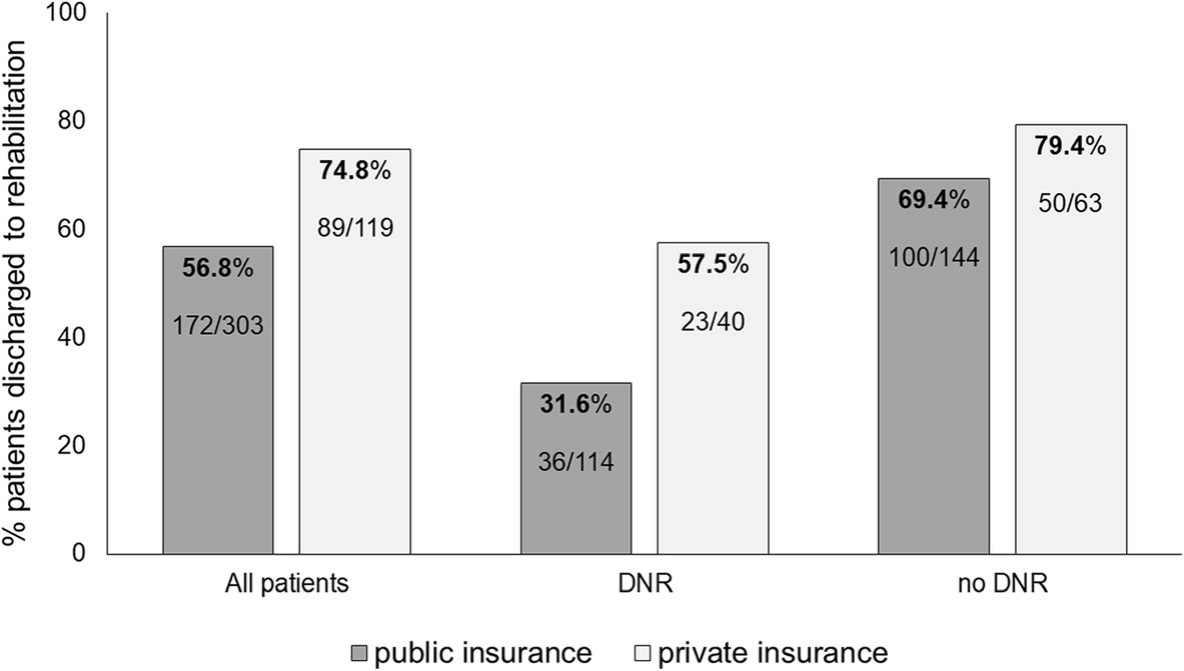
Discharge to rehabilitation versus ATC in dependence on resuscitation and insurance status

Since we found the association with DNR interesting, we performed another stepwise logistic regression including only patients with DNR order discharged to ATC or rehabilitation (regression IV, Table 3). The younger the DNR-positive patients and the lower their DOS scores, the more likely they were to be discharged into rehabilitation rather than ATC. Also, the presence of supplementary health insurance was a predictor for discharge into rehabilitation. Figure 1 illustrates the dependency between the place of discharge (ATC versus rehabilitation), the presence of supplementary health insurance and of DNR order. Patients without DNR order more often went into rehabilitation, whereas patients with DNR order more often went into ATC as compared to the overall sample. Generally, patients with supplementary health insurance more often went into rehabilitation. The effect of supplementary insurance on the place of discharge is even greater for patients refusing resuscitation than for those who agree on it (Fig. 1).

**Table 3:** Factors associated with the place of discharge for patients with DNR order

Regression IV: Discharge to ATC or rehabilitation (*n* = 128)		
Variable	Odds Ratio	95% CI
Age	0.89 ***	(0.84; 0.94)
DOS	0.73 *	(0.55; 0.98)
Supplementary health insurance	3.76 **	(1.52; 9.29)
Hosmer–Lemeshow test: *P* = 0.469		

*Note*. Stepwise logistic regression (*n* = 128). The dependent variable in this analysis is the place of discharge (ATC or rehabilitation). Significance level for inclusion in the model was *p* = .20. Predictive significance levels: * *p* < .05, ** *p* < .01, *** *p* < 0.001. Hosmer–Lemeshow test indicates that the overall model fit is good. Reference group for dichotomous independent variable: insurance – 0 = public. Reference groups for dichotomous dependent variable: rehabilitation = 1. *DOS* Delirium Observation Screening Scale

## Discussion

We identified factors associated with the place of discharge after an acute hospital stay in patients 50 years and older, namely into rehabilitation clinics or in-patient ATC units. We found that the lack of supplementary health insurance was associated with discharge into ATC rather than rehabilitation or any other place of discharge. According to Swiss healthcare experts, having supplementary health insurance is not relevant for highly qualitative and holistic healthcare, but simply provides additional conveniences [20]. Our results contradict this assessment as we find that the lack of supplementary health insurance increases the likelihood of discharge into ATC after hospital stay, which is financially less favorable than rehabilitation for inpatients. The results further illustrate that the association of having supplementary health insurance with the place of discharge prevailed despite the association of DNR status with the place of discharge (Fig. 1, Table 3).

Our finding furthermore is in line with other Swiss studies showing a higher probability for inpatient rehabilitation of stroke [21] and cancer [22] patients with supplementary health insurance. This demands further critical examination, including the assessment of whether older patients and patients with basic health insurance receiving ATC actually would have qualified for and benefitted from rehabilitation. An unintended health-relevant benefit of supplementary insurance also remains to be further examined and would be of ethical and legal relevance. Previous studies have shown that patients with lower income and a smaller social network have poorer health prognoses [23, 24]. Our data suggest that they are also less likely to receive rehabilitation if we assume that patients with low income cannot afford supplementary health insurance.

Our result that a larger social network is associated with discharge into rehabilitation rather than ATC indicates that the social network might support inpatients in expressing and reinforcing their wish for rehabilitation, thereby receiving a financially better discharge option. That social network plays a role in the discharge into rehabilitation was shown before in a British study [25]. The influence of social networks on the place of discharge might be especially disadvantageous for vulnerable groups of older patients who may lack social network, which is ethically problematic because they might not get the best possible post-acute care.

The finding that the older the patient, the more likely they are to go into ATC rather than rehabilitation was already shown in a previous analysis from our group [26]. The same study also found that male patients were more likely to be discharged to rehabilitation than female patients, which we were able to confirm when comparing discharge into rehabilitation versus all other places of discharge. That no DNR order and low DOS were associated with discharge into rehabilitation can be explained by the criteria to be admitted to rehabilitation [11]: Patients in delirium at the moment of discharge cannot fully benefit from rehabilitative interventions; and refusing resuscitation upon cardiac arrest can be seen as an indicator for a lower potential to meet rehabilitation aims.

As our results suggest that patients released to ATC are already frail, it is questionable whether the original aim to send these patients home is sustainable. Indeed, readmission rates increased since the introduction of SwissDRG and ATC [27], and studies from other settings show that frail patients are likely to be readmitted to acute care hospitals [28]. The effectiveness of ATC should, therefore, be reconsidered.

### Limitations

This study has several limitations. Because of the explorative nature of this study, the sample size for group comparison is a limiting factor, and we recommend verifying our results with larger sample sizes, including a greater number of hospitals and hospitals from different regions. Furthermore, we applied a medical social network sum score that was based on previously validated scores, but its application to medical records has not been validated. The social support sum score furthermore does not reflect the patient’s real social network, since it is based on medical records and not a self-assessment tool. However, the fact that social contacts are stated in the patient’s medical record clearly indicates that they are involved in the patient’s healthcare or daycare management.

## Conclusion

Our study findings are derived from data originating from one Swiss hospital. Nevertheless, the results still have international relevance, because transitional care units after acute hospital stays are increasingly important in international public health settings [4–6]. While previous studies showing similar results were diagnosis-specific [21, 22, 25], our results are from patients with different diagnoses and hence reveal that the association of the identified factors with the place of discharge is independent of medical diagnoses. Having supplementary health insurance and the social network size should not be influential for the place of discharge when looking at the indications for both ATC and rehabilitation. Moreover, rehabilitation is more favorable for patients than ATC from a personal financial perspective, and our findings suggest that patients with an already weaker financial background (that are not having supplementary health insurance, presumably because they cannot afford it) are burdened with additional costs when going to ATC. To address the here-mentioned issues, we suggest standardizing discharge into post-acute care facilities making it more independent from financial aspects and social networks. Social workers, in collaboration with doctors and nurses, play a leading role in implementing such procedures in acute care hospitals, but the incentive and framework should come from the public health sector.

## Additional file

Additional file 1: **Table S1.** Places of Discharge (*n* = 659). Descriptive statistics on place of discharge in the data set. (PDF 91 kb)

## Abbreviations

ATC: Acute and Transitional Care
DNR: *Do Not Resuscitate*
DOS: Delirium Observation Scale
KVG: Federal Act on Health Insurance
LOS: Length of hospital stay
SwissDRG: Diagnosis-Related Groups in Switzerland
