# Supplementary material for: Factors associated with post-acute discharge location after hospital stay: a cross-sectional study from a Swiss hospital
Source: BMC Health Serv Res. 2019 May 8;19:289. doi: 10.1186/s12913-019-4101-6 (PMC6505070; doi:10.1186/s12913-019-4101-6)
Supplement: Supplementary file 1 — Table S1. Places of Discharge (n = 659). Descriptive statistics on place of discharge in the data set. (PDF 91 kb) [file 12913_2019_4101_MOESM1_ESM.pdf]

Supplementary

Supplementary table. Places of Discharge (n=659).

**Place of Discharge**	**Number of Patients (Percentage)**
Rehabilitation	262 (39.8%)
Acute and transitional care	161 (24.4%)
Home (with or without help)	113 (17.1%)
Nursing home	41 (6.2%)
Acute geriatric hospital	29 (4.4%)
Curative institutions	16 (2.4%)
Others	37 (5.6%)

Note. Due to rounding percentages may not add up to 100%. Total number of patients was n=660, but for one patient information on place of discharge was not available from the medical record.
